# Numerical Analysis of Curing Residual Stress and Deformation in Thermosetting Composite Laminates with Comparison between Different Constitutive Models

**DOI:** 10.3390/ma12040572

**Published:** 2019-02-14

**Authors:** Jianfeng Dai, Shangbin Xi, Dongna Li

**Affiliations:** 1 State Key Laboratory of Advanced Processing and Recycling of Non-Ferrous Metal Material, Lanzhou University of Technology, Lanzhou 730050, China; 2 School of Aerospace Engineering, Beijing Institute of Technology, Beijing 100081, China; binbinqq136com@163.com; 3 School of Mechanical Engineering, Lanzhou Jiaotong University, Lanzhou 730070, China; lidongna9895@163.com

**Keywords:** thermosetting composite, curing process, multi-physics coupling, residual stress, deformation

## Abstract

A multi-physics coupling numerical model of the curing process is proposed for the thermosetting resin composites in this paper, and the modified “cure hardening instantaneously linear elastic (CHILE)” model and viscoelastic model are adopted to forecast residual stress and deformation during the curing process. The thermophysical properties of both models are evolved in line with temperature and degree of cure (DOC). Accordingly, the numerical simulation results are improved to be more accurate. Additionally, the elastic modulus of the materials is calibrated to be equal to the modulus of viscoelastic relaxation by a defined function of time in the CHILE model. Subsequently, this work effectuates the two proposed models in a three-dimensional composite laminate structure. Through comparing the two numerical outcomes, it is customary that the residual stress and deformation acquired by the modified model of CHILE conform to those ones assessed through adopting the viscoelastic model.

## 1. Introduction

By virtue of advantaged properties in mechanical aspects, the thermosetting resin composites have been extensively adopted in numerous fields, including civil infrastructure, ship industries, and the aerospace industry. However, during the manufacturing process of the composites, several phenomena will consequently generate the residual stress and deformation of the composite structures [1,2,3], and therefore decrease the mechanical properties taken on by the composites. These phenomena include material relaxation or degradation, chemical shrinkage, thermal expansion, and inherent anisotropy. In this regard, evaluating the residual stress and deformation in the design and manufacturing of the composite materials is imperative. The procedure of trial-and-error method is the industrial method for estimating the partial deformation. This method is costly, time-consuming, and inaccurate. Comparatively, the simulation of finite element analysis (FEA) is a method capable of accurately, simply, and effectively estimating the residual stress and deformation.

Up until now, the method of FEA has been extensively adopted for composites in the curing process [4,5,6,7,8,9,10,11,12], and diverse models have been developed to forecast the stress and deformation that arises from uneven degree of cure (DOC) and temperature [13,14,15,16,17,18,19,20]. Numerical models based on finite element methods (FEM) have been used to research the mechanisms generating residual stresses [4]. Because of the appropriate composite models, inclusive of the foregoing factors, the mechanical response evolution is able to be well described by the stress and deformation taken on by the structures of composite in the period of curing. Among the mentioned models, the viscoelastic model, the cure hardening instantaneously linear elastic (CHILE) model, and the elastic model are frequently adopted to predict how the residual stress will develop in composites. These three models take on distinctive strengths and weaknesses, as well as the scope of adoption. Admittedly, the polymer takes on viscoelasticity in the period of curing, particularly in the heat-up period and the hold period. The elastic model is able to be merely adopted to forecast how the internal stress develops in the cool-down period, but neglects how the stress develops over the course of curing [21,22]. In this regard, given that the variation of elasticity modulus is deemed by the model as a function of DOC and temperature in the course of the cure period, the CHILE model is the model proposed by some researchers to assess the deformation and residual stress of the composite laminates effectively [16,23,24,25]. Yet, the CHILE method remains unable to elucidate the viscoelastic performance taken on by the composites in the course of the cure period, for instance, the relaxation of stress, causing the deficient generality and validity taken on by an approximation of CHILE. Accordingly, composite models with n Maxwell elements, related to viscoelasticity on the basis of a generalized Maxwell model, are prioritized by other scholars to indicate the features related to viscoelasticity taken on by the composites during the course of the cure period [17,26,27]. Through incorporating FEA and this model, the internal stress and deformation taken on by the composite laminates in the entire course of the curing period can be forecast accurately and effectively [28]. This model fails to be extensively adopted, arising from difficulty in numerical implementation, tedious runtimes, and the lacked experiment evidence, even though the current simplified integration approaches are adopted, or models related to viscoelasticity takes on evident strengths for making the composite more accurate [29]. To effectuate the model readily through factoring in the performance related to viscoelasticity, a modified CHILE model was developed by several researchers. This model defines a particular time function for the correction of the modulus related to elasticity through establishing it by approaching the relaxation modulus related to viscoelasticity and elasticity [23,30]. Through adopting the proposed model to forecast curing process-induced residual stress and deformation taken on by the composites, the outcomes of simulation conform to the results acquired through adopting the model related to viscoelasticity effectively.

The AS4/3501-6 carbon fiber epoxy resin, the viscoelastic model, and the modified CHILE model are adopted in this paper in a 3D structure of composite laminate to acquire the process-induced residual stress and deformation in the course of curing cycle of the composite laminates. On this basis, this work draws the comparison between the outcomes of simulation acquired through adopting two models. Additionally, this work references the current outcomes of simulation developed by others. Eventually, in line with practical requirements and conditions, the work is able to select the appropriate model following the comparative outcome.

## 2. Control Equation

### 2.1. Heat Transfer Equation

In the course of cure period of the thermosetting resin composites, the conduction and generation of heat originates from heat being internally nonlinear in the curing of resin. In this regard, sources of internal heat and external heat flux are involved in the model of transient heat transfer. The model of transient heat transfer is based on Fourier’s heat conduction equation, and can be described as [1,2]:

(1) $$\rho_{c}C_{pc}\frac{\partial T}{\partial t}=\frac{\partial }{\partial x}\left(k_{x}\frac{\partial T}{\partial x}\right)+\frac{\partial }{\partial y}\left(k_{y}\frac{\partial T}{\partial y}\right)+\frac{\partial }{\partial z}\left(k_{z}\frac{\partial T}{\partial z}\right)+\frac{\partial Q}{\partial t},$$

where Q is the internal heat source; T is the instant temperature taken on by composite materials at time t, and t refers to the absolute time; $k_{x}$, $k_{y}$ and $k_{z}$ denote the anisotropic thermal conductivities taken on by composite materials in the directions of x, y, and z; $C_{pc}$ and $\rho_{c}$ refer to the specific heat and density of the composites. The internal heat source Q is the instantaneous heat generated by the cross-linking polymerization of the resin, which is governed by:

(2) $$\frac{\partial Q}{\partial t}=\rho_{r}\left(1-v_{f}\right)H_{R}\frac{d\alpha }{dt},$$

where $H_{R}$ is the total release heat during curing; dα/dt and α represent the curing rate of the resin matrix and DOC, respectively; $v_{f}$ represents the fiber volume fraction taken on by composite materials; $\rho_{r}$ is the density of the resin matrix.

### 2.2. Chemical Reaction

The standard differential scanning calorimetry (DSC) counts as the common experiment-based method adopted to study the curing-related kinetics taken on by the thermoset resin composites [31,32]. In the course of the curing period, the resin matrix appears as a bulk of complicated chemical reactions with the development of complicated cross-linked structures and a massive release of heat. In this regard, to shed light on the curing course via a rate function, this work employs a phenomenological model relative to the curing-related kinetics. For 3501-6 epoxy resin, the rate of conversion dα/dt is given by the following equations [1,2,33]:

(3) $$\{\begin{array}{l} \frac{d\alpha }{dt}=\left(K_{1}+K_{2}\alpha \right)\left(1-\alpha \right)\left(0.47-\alpha \right)\;\alpha \leq 0.3\\ \frac{d\alpha }{dt}=K_{3}\left(1-\alpha \right)\;\alpha >0.3 \end{array}\;\;\;,$$

where $K_{i}(i=1,2,3)$ indicates the curing rate constants, which can be given by the Arrhenius functions as follows:

(4) $$K_{i}=A_{i}\exp\left(\frac{-∆E_{i}}{RT}\right),\;\left(i=1,2,3\right)$$

where $A_{i}$ are the Arrhenius constant; $∆E_{i}$ are the activation energies, and R is the gas constant. Table 1 lists the parameters of curing-related kinetics of 3501-6 epoxy resin.

### 2.3. Thermo-Physical Properties

The instantaneous heat conduction model and the solidification kinetics model can effectively simulate the thermochemical process during solidification. This means that the thermo-physical properties of composites and their composition can accurately predict the solidification behaviors of composite materials which mainly contain thermal conductivity, specific heat, and density. Evidently, this temperature and the change in the thermal physical properties of DOC are considered. The thermal properties of AS4 carbon fiber and 3501-6 epoxy resin are listed in Table 2. The thermal physical properties of composites are able to be obtained through adopting the mixture rule in Equations (A1)–(A4) proposed in Appendix A. Viscoelastic model.

Currently, the viscoelastic model, even if there are some disadvantages (such as running time consumption and complex formulas) [26,27,28,31,33], remains the most accurate method to forecast the viscoelastic behavior of resins and thermosetting materials. This model factors in the time, temperature, and the change of DOC, a Boltzmann superposition principle. Under the method of time–temperature superposition, the stress of viscoelastic materials is represented by the constitutive function below, in the form of genetic integral [26,27,31]:

(5) $$\underline{\sigma }\left(t\right)=\int_{0}^{t}\underline{\underline{C}}\left(\alpha ,T,t-\tau \right)\frac{d}{d\tau }\left[{\underline{\varepsilon }}_{tot}\left(\tau \right)-{\underline{\varepsilon }}_{tc}\left(\tau \right)\right]d\tau ,$$

where the free thermo-chemical strain ${\underline{\varepsilon }}_{tc}$ is defined as

(6) $${\underline{\varepsilon }}_{tc}={\underline{\varepsilon }}_{th}+{\underline{\varepsilon }}_{ch}=\underline{\phi }∆T+\underline{\varphi }∆\alpha ,$$

where $\underline{\varphi }$ and $\underline{\phi }$ refer to the coefficient of shrinkage expansion (CSE) and thermal expansion (CTE) in each orientation. Thermal expansion strain, ${\underline{\varepsilon }}_{th}$, and cure shrinkage strain, ${\underline{\varepsilon }}_{ch}$, are contained as free thermo-chemical strain. ${\underline{\varepsilon }}_{tot}$, τ and t refer to total strain, past time, and current time. $\underline{\underline{C}}$ denotes the relaxation stiffness matrix as functions of DOC, temperature, and time. This work adopts single underlines and double underlines to indicate the vectors and matrices. This work can denote Equation (5), as below, as being in line with the features taken on by thermorheologically simple materials at a constant DOC:

(7) $$\underline{\sigma }\left(t\right)=\int_{0}^{t}\underline{\underline{C}}\left(\xi \left(t\right)-\xi^{\prime }\left(\tau \right)\right)\frac{d}{d\tau }\left[{\underline{\varepsilon }}_{tot}\left(\tau \right)-{\underline{\varepsilon }}_{tc}\left(\tau \right)\right]d\tau ,$$

in which:

(8) $$\xi \left(t\right)=\int_{0}^{t}\frac{dt^{\prime }}{a_{T}\left[\alpha_{ref},T\left(t^{\prime }\right)\right]},\;\xi \left(\tau \right)=\int_{0}^{\tau }\frac{dt^{\prime }}{a_{T}\left[\alpha_{ref},T\left(t^{\prime }\right)\right]},$$

where $\alpha_{T}$ is the shift factor which allows for time–temperature superposition, $\alpha_{\mathrm{ref}}$ references DOC, and ξ(t) and ξ(τ) indicate the past reduced time and current reduced time, respectively.

The relaxation modulus of a viscoelastic material is denoted via a series of Prony, as shown below, in light of the generalized Maxwell model with *n* Maxwell elements in parallel [26]:

(9) $$E\left(\alpha ,\xi \right)=E^{\infty }\left(\alpha \right)+\left[E^{u}\left(\alpha \right)-E^{\infty }\left(\alpha \right)\right]\sum_{i}^{n}W_{i}\left(\alpha \right)\exp\left[\frac{-\xi \left(\alpha ,T\right)}{\tau_{i}\left(\varepsilon \right)}\right]$$

Here, $W_{i}$ is weigh factor, and $E^{u}$ and $E^{\infty }$ indicate the adequately unrelaxed and relaxed modulus. The non-relaxation materials properties and proposed relaxation modulus, inclusive of CSE, CTE, Poisson’s ratio, shear modulus, and modulus related to elasticity, can be adopted to acquire the performance relative to viscoelasticity taken on by the anisotropic composite laminates precisely in the light of the micro-mechanics theory [1]. Table 3 lists the unrelaxed mechanical properties (namely, mechanical properties relative to elasticity) of AS4/3501-6 prepreg constituents. Furthermore, this work is able to acquire the mechanical properties taken on byAS4/3501-6 prepreg through adopting micromechanical Equations (A.8)–(A.16), as exhibited in Appendix A.

### 2.4. Modified CHILE Model

The CHILE model continues to be the essential, critical elasticity-related model. A modified CHILE model is able to be acquired to forecast residual stress and deformation of the viscoelastic materials as the work adopts an equivalent modulus corresponding to the relaxation modulus, related to viscoelasticity at certain frequencies or a specific time, though the CHILE model fails to shed light on the state of the polymer being in the viscoelastic regime in the course of the cure period. The acquired outcomes turn out to be nearly equal to those forecasted through adopting the viscoelasticity-related method. The work can denote the integral form of the CHILE constitutive function as:

(10) $$\underline{\sigma }\left(t\right)=\int_{0}^{t}E\left(\alpha ,T\right)\frac{d}{d\tau }\left[{\underline{\varepsilon }}_{tot}\left(\tau \right)-{\underline{\varepsilon }}_{tc}\left(\tau \right)\right]d\tau ,$$

where E indicates the instant elasticity-related modulus that is estimated. And more notably, the temperature and DOC change along with time in the course of the cure cycle. Thus, the constitutive equation has the form of the following equation:

(11) $$\underline{\sigma }\left(t\right)=\int_{0}^{t}E\left(\tau \right)\frac{d}{d\tau }\left[{\underline{\varepsilon }}_{tot}\left(\tau \right)-{\underline{\varepsilon }}_{tc}\left(\tau \right)\right]d\tau .$$

Evidently, Equations (7) and (11) shall be identical ones as the function below is effectuated [30]:

(12) $$E\left(\tau \right)=\underline{\underline{C}}\left[\xi \left(t\right)-\xi^{\prime }\left(\tau \right)\right].$$

Here, we can see that Equation (12) seems odd, because the part to the right of the equal sign is a relaxation modulus related to both t and τ, whereas the left-hand side is a modulus related to elasticity, which is merely an equation of τ. This function shall be expounded in the paragraphs of this section below. To assess Equation (11), modulus related to elasticity E, at any integration time τ, is required to be assessed to meet Equation (12), i.e., the modulus related to elasticity, and E can be defined as the value of the relaxation modulus at time τ, $\underline{\underline{C}}$, at an appropriate undetermined time, $t_{e}$, under the DOC and temperature at time τ. Accordingly, this work can denote Equation (12) as:

(13) $$E\left[T\left(\tau \right),\alpha \left(\tau \right)\right]=\underline{\underline{C}}\left[t_{e},T\left(\tau \right),\alpha \left(\tau \right)\right],$$

where $t_{e}$ indicates an undetermined and appropriate time. It is noteworthy that the DOC and temperature are functions of τ for the relaxation modulus, $\underline{\underline{C}}$, and the modulus related to elasticity. The work can denote the relaxation modulus at time $t_{e}$ as below, in light of the principle of time–temperature superposition:

(14) $$\underline{\underline{C}}\left[t_{e},T\left(\tau \right),\alpha \left(\tau \right)\right]=\underline{\underline{C}}\left[\frac{t_{e}}{a_{T}\left(\tau \right)}\right].$$

Evidently, the function below is required for Equations (12)–(14):

(15) $$\underline{\underline{C}}\left[\frac{t_{e}}{a_{T}\left(\tau \right)}\right]=\underline{\underline{C}}\left[\xi \left(t\right)-\xi^{\prime }\left(\tau \right)\right].$$

A necessary and sufficient condition for Equation (15) is given for Equation (16):

(16) $$\frac{t_{e}}{a_{T}\left(\tau \right)}=\xi \left(t\right)-\xi^{\prime }\left(\tau \right).$$

Substituting Equation (8) into Equation (16), the function can be reorganized below:

(17) $$\frac{t_{e}}{a_{T}\left(\tau \right)}=\int_{\tau }^{t}\frac{dt^{\prime }}{a_{T}\left[\alpha_{ref},T\left(t^{\prime }\right)\right]}.$$

Meanwhile, the determination of $t_{e}$ is counted as a problem that is critically difficult. To reckon with this, the integral in the Equation (17) falls into two parts: the cool-down stage and the hold stage.

(18) $$\frac{t_{e}}{a_{T}\left(\tau \right)}=\int_{\tau }^{t_{f}}\frac{dt^{\prime }}{a_{T}\left[\alpha_{ref},T\left(t^{\prime }\right)\right]}+\int_{t_{f}}^{t}\frac{dt^{\prime }}{a_{T}\left[\alpha_{ref},T\left(t^{\prime }\right)\right]},$$

where $t_{f}$ denotes the time at the preliminary cool-down stage and the end of the hold stage. The second integral belongs to the cool-down regime, whereas the first one belongs to the hold regime. Accordingly, this work can assess the time $t_{e}$ as below through deducing the two integrals [30]:

(19) $$t_{e}=\frac{1}{2}\left(t_{m}-\tau \right)\left[1+\frac{a_{T}\left(\tau \right)}{a_{T}\left(t_{m}\right)}\right]+\left(t_{f}-t_{m}\right)\cdot \frac{a_{T}\left(\tau \right)}{a_{T}\left(t_{f}\right)}+\frac{\log\left(e\right)}{c\left(\alpha_{ref}\right)\eta }\cdot \frac{a_{T}\left(\tau \right)}{a_{T}\left(t_{f}\right)},$$

Eventually, the state of stress for the composites is able to be accurately acquired through adopting Equations (10), (13), and (19).

## 3. Numerical Formulation

Residual stress strongly effects the properties of composite parts manufactured with thermosetting polymers. The residual stress and deformation for the composites was simulated through adopting the viscoelastic approach and the modified CHILE method. This work draws a comparison between the two sets of outcomes, and also references White and Kim’s research [34,35]. That it is conducive to anatomize the simulation of optimization for the evolution of residual stress and deformation.

**Figure 1:**
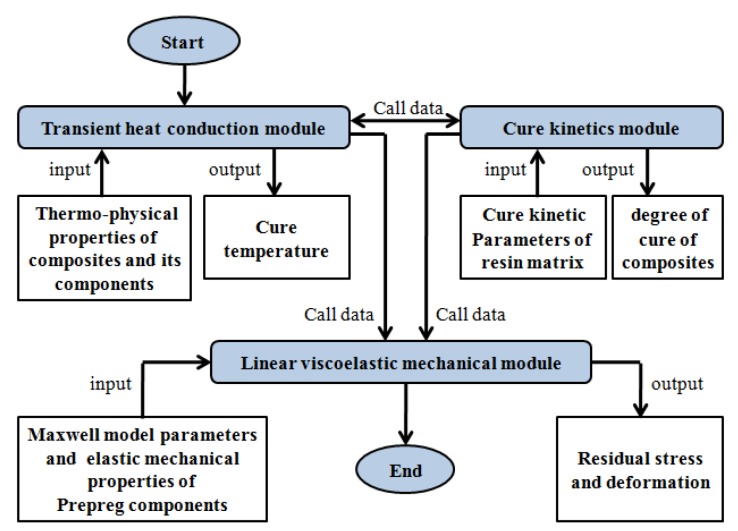
Put forward process to simulate residual stress and deformation under the viscoelastic model.

Two models of simulation were encompassed by three coupled modules; mechanical modules, instant heat conduction, and cure kinetics. Given that the output data of some modules count as the input data of others, the order of loading these modules is of critical significance in the model. Figure 1 presents flow of the viscoelastic model for the simulation of residual stress and deformation, in light of the loading order of these modules. Instant heat conduction, in light of Equation (1), is loaded, and it is the first loading module that provides curing temperature for other modules and counts as the critical basis in curing process. The cure kinetics is loaded on the second module, adopting Equation (3), and offering DOC for the mechanical module. As DOC and temperature are correlated, the modified CHILE model is in light of Equation (10), and the viscoelastic model adopts Equation (5). At last, the mechanical module is loaded. It is noteworthy that, as several thermo-physical properties transmit to heat conduction module require DOC captured from the second module, the first and second modules call data with each other in each time increment.

In COMSOL Multiphysics (Version 4.3b), such a process should be a repeated iteration of each increment until the desired precision is reached (for this article we selected 0.001). In this respect, when the thermodynamic analysis of coupling is performed, that is, the local error, the error is within one time. The global error is the estimate of the sum of local errors, in excess of, or under, the sum of local errors. Therefore, after the proper accuracy test, the calculation precision can be improved effectively.

This work adopts some normal COMSOL Multiphysics modules to realize the two models of process. This work effectuates the instant heat conduction module through introducing the thermo-physical property parameters presented in Table 2, and through adopting the “Heat Transfer” Application Module. Additionally, in this Application Module, this work establishes the “Heat Source” term through factoring in chemical heat release, bound by the DOC (Equation (2)). In this work we implemented the module of Cure kinetics through adopting the “Coefficient format PDF” Application Module, taking on user-defined function. The general function of this module is defined below:

(20) $$e_{a}\frac{\partial^{2}u}{\partial t^{2}}+d_{a}\frac{\partial u}{\partial t}+\nabla \cdot \left(-c\nabla u-\alpha u+\gamma \right)+\beta \cdot \nabla u+au=f,$$

where f is the source term; c, a, $e_{a}$, $d_{a}$, α, β and γ are all coefficients; u and t are dependent variable and instant time, respectively. The cure rate functions (Equations (3) and (4)) are established in Equation (20) in this paper through adopting the appropriate coefficients. The DOC can be acquired by following the cure kinetic parameters listed in Table 1. Eventually, this work adopts the “Viscoelastic Material” item to effectuate the mechanical module in viscoelastic model in line with Equation (5). Such item is selected from “Structural Mechanics” Application Module. In the “Long-Term Elastic Properties” of the “Domain Selection”, “Young’s modulus and shear modulus” item is specified as well as the defined relaxation modulus, which is set in the “Generalized Maxwell Model” tab, presented from Equation (9), and the material parameters listed in Table 3 are accessed to the related fields.

The mechanical module in the modified CHILE model is implemented in the “Elastic Mechanics” item chosen from “Structural Mechanics” Application Module in the light of Equation (11). In line with Equations (13) and (19), time variable $t_{e}$ is set to participate in the definition of modulus related to elasticity for obtaining an equivalent modulus that corresponds to the viscoelastic relaxation modulus.

## 4. Material Model Verification

**Figure 2:**
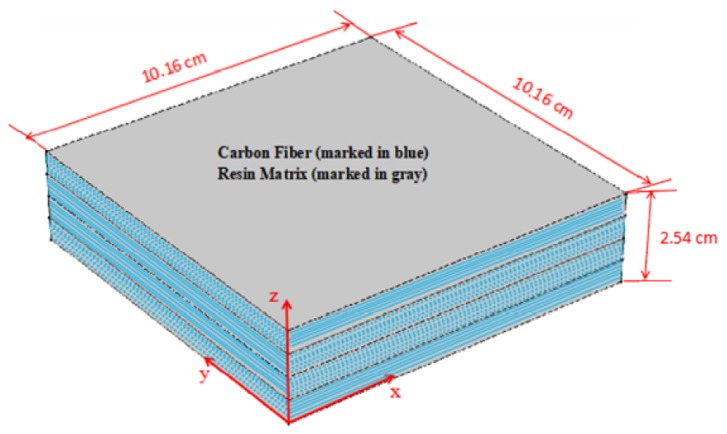
Schematic of the composite laminates.

To judge the accuracy and efficiency of the two proposed models for simulation, this paper adopts a same-thickness-plies orthotropic composite laminate and a stacking sequence of [0°/90°/90°/0°] (as presented in Figure 2) as an instance, with the size ascertained as 10.16 cm × 10.16 cm × 2.54 cm. An ideal contact interface is set between layers, that is, the natural boundary (Newman boundary) condition. The work adopts the AS4/3501-6 prepreg encompassing AS4 fibers pre-impregnated with 3501-6 epoxy resin as the major material, where the fiber volume fraction taken on by composite materials reaches 50%. The cross-ply laminate of AS4/3501-6 is acquired through introducing the related parameters of the material to the proposed simulation models.

**Figure 3:**
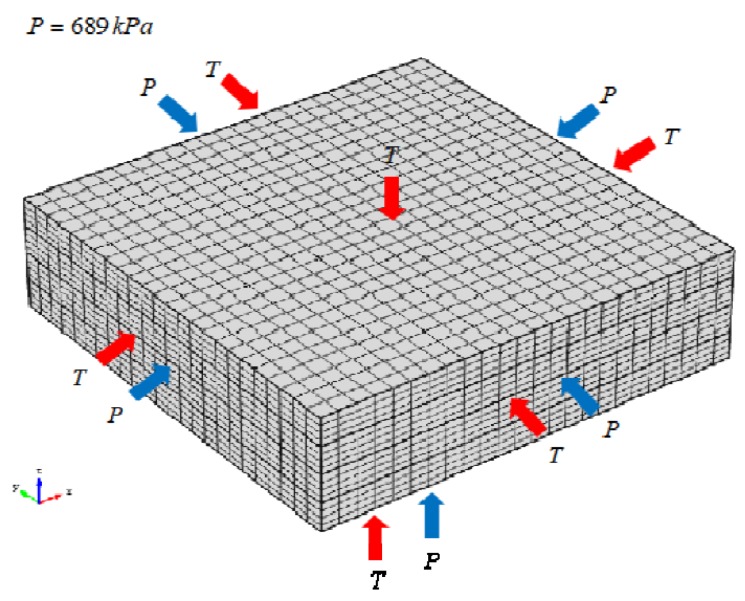
Meshing and boundary conditions of the composite laminates.

The period of curing recommended from the manufacturer is used in these simulations which contains five stages: first heat-up period with a ramp rate of 2.5 °C/min from 25 °C to 116 °C, first 1-h hold period at 116 °C, second heat-up period with the same ramp rate to 177 °C, second 2-h hold period at 177 °C, eventually, cool-down period with a ramp rate of −2.5 °C /min from 177 °C to 25 °C. Two boundary conditions, heat transfer and mechanical pressure, are used to simulate the tool-part interfaces, which can be set in the instant heat conduction module and the mechanical module, respectively. The mold heating temperature is defined on the outside surface of the composite laminates, while the pressure loaded on the three planes is kept during the whole cure cycle (as presented from Figure 3).

In addition, it is necessary to verify the mesh to obtain an appropriate mesh, the shape and refinement of which impact on the convergence and accuracy of simulation. In this model, a hexahedral mesh is employed as refined mesh (as presented in Figure 3). The number of elements per layer is 2304, and the total number is 9216.

## 5. Results and Discussion

### 5.1. Thermo-Chemical Analysis

**Figure 4:**
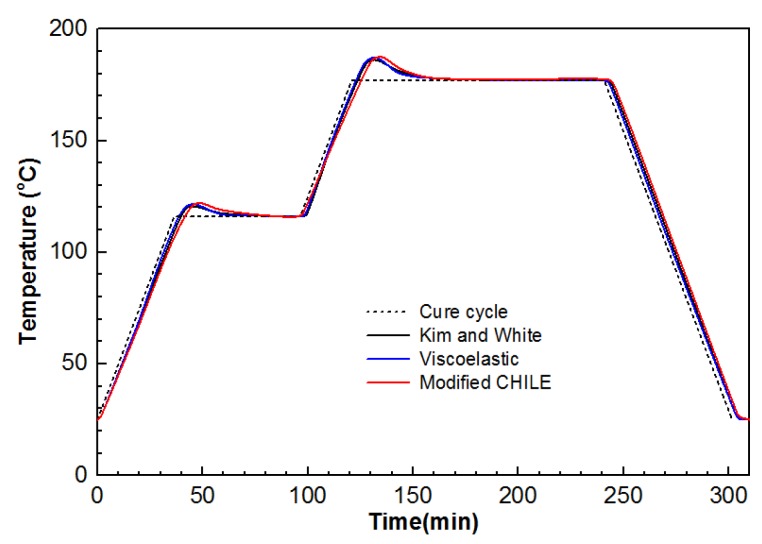
Temperature development in central point (5.08, 5.08, 1.27) of the composite laminate under from the modified cure hardening instantaneously linear elastic (CHILE) and viscoelastic models.

**Figure 5:**
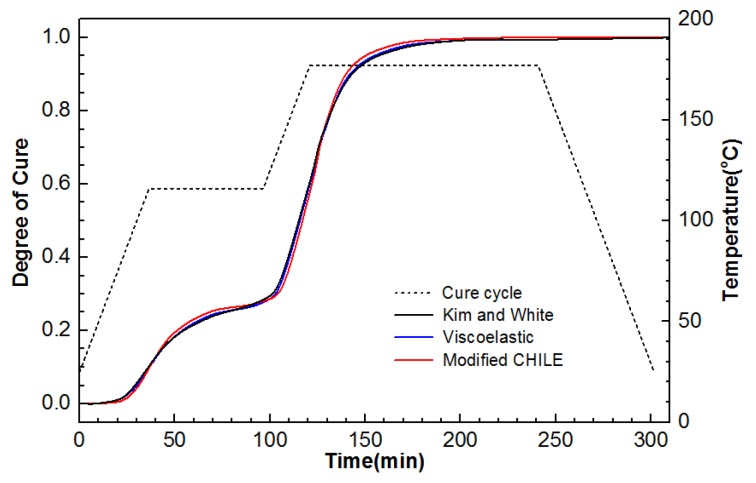
Degree of cure (DOC) development in central point (5.08, 5.08, 1.27) of the composite laminate under manufacture recommended cure cycle from the modified CHILE and viscoelastic models.

**Figure 6:**
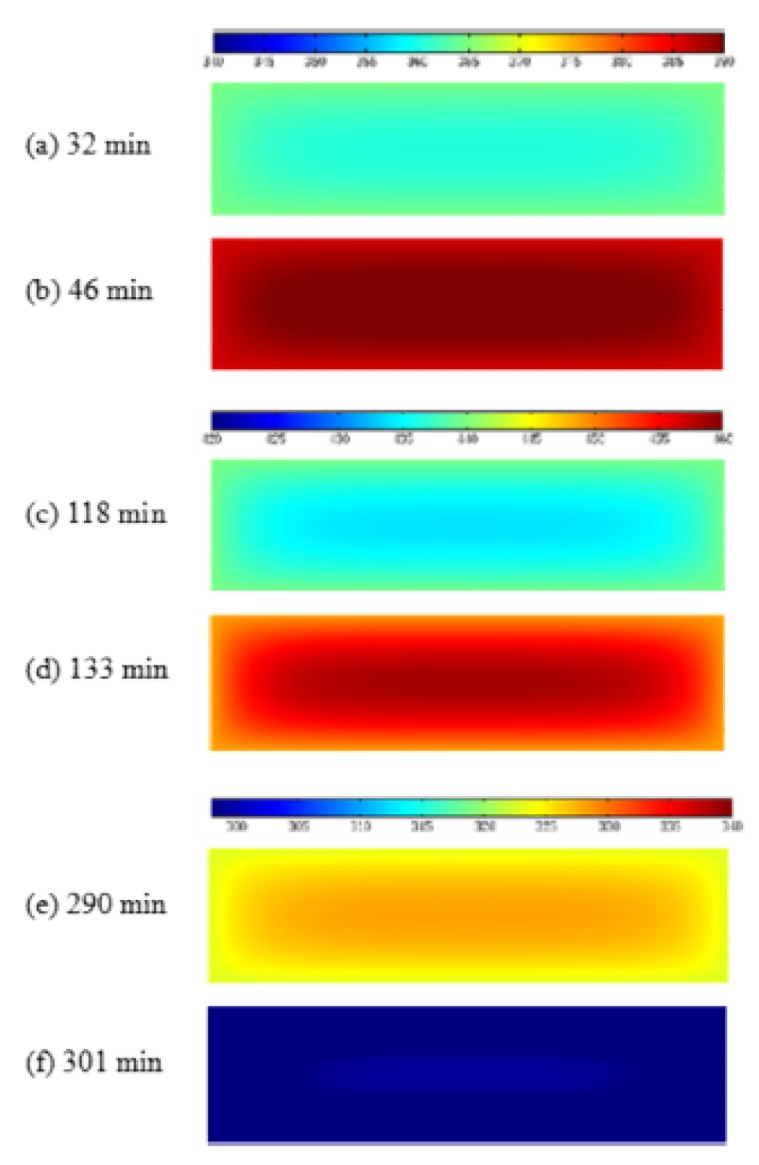
Distribution of temperature on the cross section at x = 5.08 in the course of curing from the viscoelastic model. (**a**) 32 min; (**b**) 46 min; (**c**) 118 min; (**d**) 133 min; (**e**) 290 min; (**f**) 301 min.

The temperature and DOC in central point (5.08, 5.08, 1.27) for the laminate between the modified CHILE model and the viscoelastic model are compared in Figure 4 and Figure 5 in the case of recommended period of curing for manufacture. The work also exhibits the other outcome provided by Kim and White [34]. It is noteworthy that the laminate is centered (5.08, 5.08, 1.27) between layer 3 and 2. The center temperature goes under the room temperature in the first heat-up period, (period of curing) as the surface is heated first (as presented from Figure 6a), mean time, the cross-linking rate of resin is slow. The temperature at the center rises sharply with the first peak at 46 min in the vicinity of 121 °C (as presented in Figure 6b) in the following hold period, while the cross-linking rate accelerates as the outcome of heat conduction and reaction heat accumulation. In the second heat-up period, the center temperature remains lower than the room temperature, arising from the low coefficient of heat conduction and heat transfer lag (as illustrated in Figure 6c), but the cross linking is more intensive, and the reaction rate increases significantly. In the second holding stage, arising from the internal chemical heat release and the accumulation of heat conduction of the model, high temperature area transfers to internal and the center temperature reaches the maximum temperature peak of about 121 °C at 133 min (as presented in Figure 6d). Eventually, the center temperature of the model is gradually close to room temperature (as presented in Figure 6e,f), and the DOC nearly tends to 1. The simulation results of the two models are identical and coincide with those attained in Kim and White’s study. It indicates that the heat conduction and the cure kinetics modules are quite qualified to simulate thermo-chemical response for the composite laminate during the cure cycle.

### 5.2. Residual Stress Analysis

**Figure 7:**
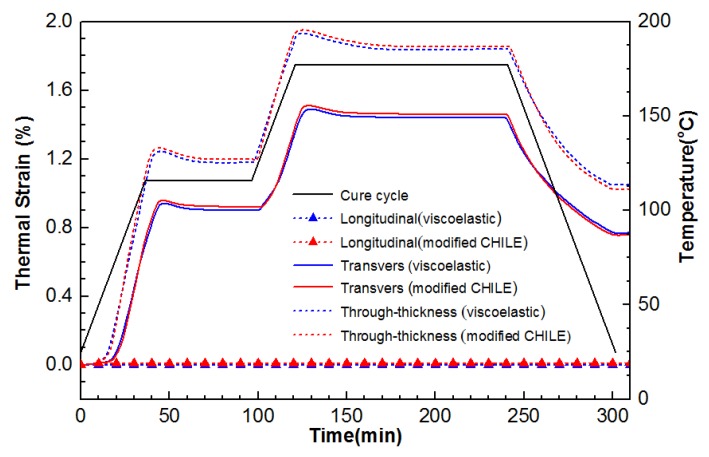
Comparison of thermal strain in various orientations at the point (5.08, 0, 1.27) from the modified CHILE and viscoelastic models.

**Figure 8:**
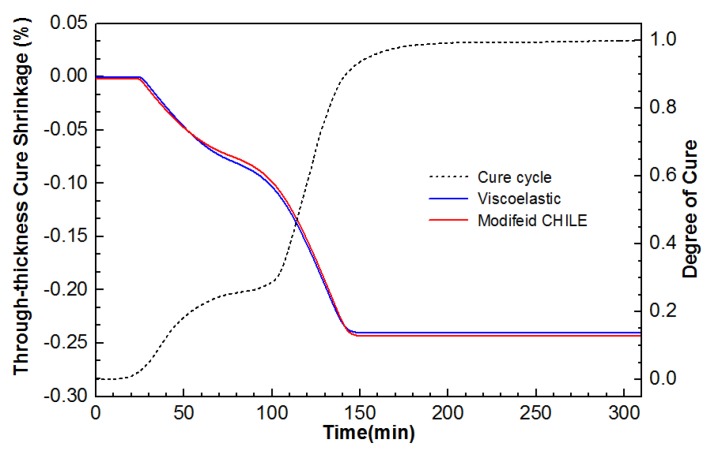
Comparison of cure shrinkage in the through-thickness orientation from the modified CHILE and viscoelastic models.

The major factor leading to residual stress generation is the cure shrinkage, i.e., the chemical shrinkage and thermal strains arising from CTE (Equation (6)). Figure 7 shows the altered state of thermal strain in different directions at the points 5.08, 0, and 1.27 for the composite laminate in the course of cure cycle. Thermal strain in the longitudinal direction is negligible, while those in through-thickness and transverse directions change noticeably, particularly during two heat-up periods. Additionally, the modified and viscoelastic CHILE models are acquired to well conform to each other. In Figure 8, the two simulation models perform well in estimating cure shrinkage from the simulated DOC in the course of cure cycle. Arising from the lateral pressure, the cure shrinkage in the through-thickness direction is evident, whereas the cure shrinkage in x and y directions can be neglected. As indicated, the cure shrinkage in the through-thickness direction computed by the modified CHILE model is in excess of that acquired by the viscoelastic model.

**Figure 9:**
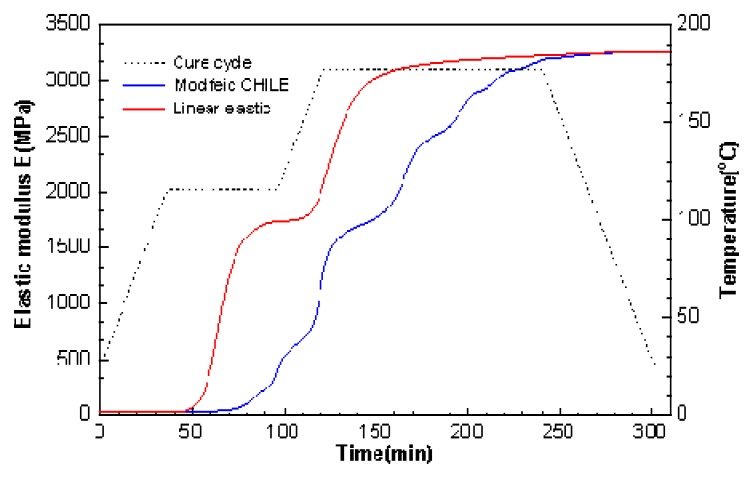
Comparison of elastic modulus in through-thickness orientation in the course of cure cycle from the linear elastic model and the modified CHILE model at constant frequency 0.1 Hz.

Figure 9 shows the simulated outcomes of modulus related to elasticity development, acquired by the linear elastic model [36] and the modified CHILE model in through-thickness direction in the course of cure cycle. Obviously, the modulus related to elasticity, acquired by the modified CHILE approach, increases slowly during curing and continues growing during the cool-down period. This outcome is contrary to the one estimated by the linear elastic model.

**Figure 10:**
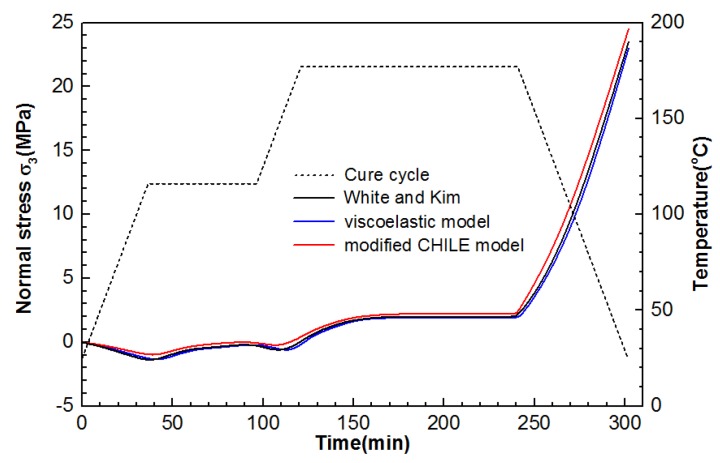
Interlaminar normal stress $\sigma_{3}$ development at the point (5.08, 0, 1.27) of the composite laminate under manufacture recommended cure cycle from the modified CHILE and viscoelastic models.

**Figure 11:**
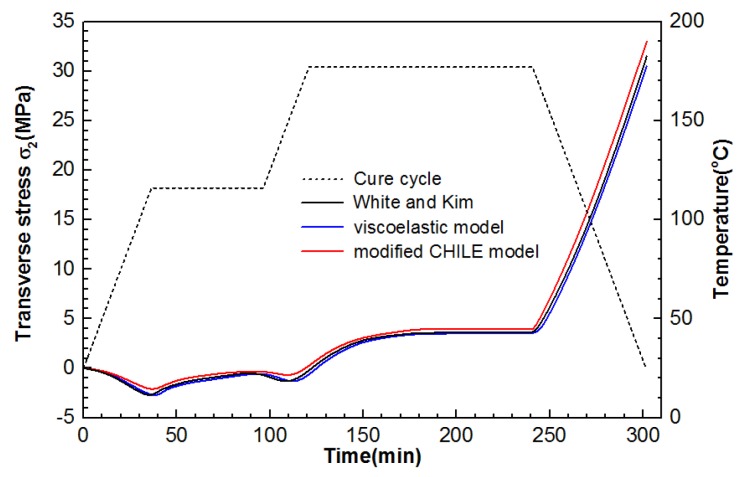
Stress $\sigma_{2}$ development in the $0^{{}^{\circ}}$ ply at x = 5.08 and y = 5.08 of the composite laminate under manufacture recommended cure cycle from the modified CHILE and viscoelastic models.

**Figure 12:**
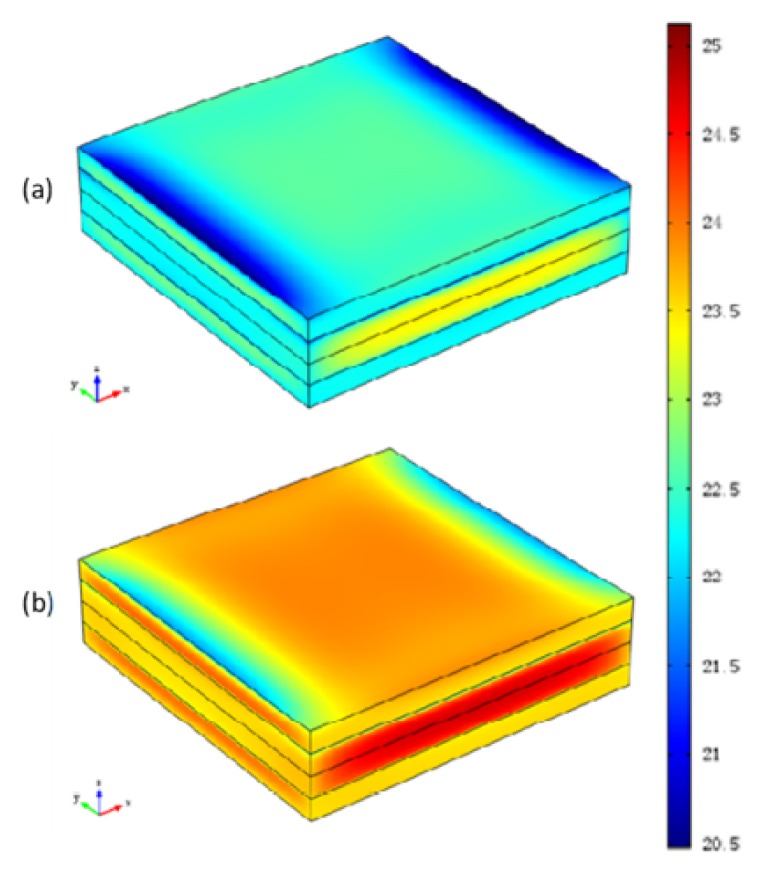
Contours of the von Mises stresses from (**a**) Viscoelastic model, (**b**) Modified CHILE model.

To prove whether the stress constitutive functions (Equations (5) and (10)) are feasible and accurate in the two proposed models, this work acquired transverse stress $\sigma_{2}$ in the 0° ply at x = 5.08 and y = 5.08 of the laminate and the development of interlaminar normal stress $\sigma_{3}$ at the points 5.08, 0, and 1.27, presented from Figure 10 and Figure 11. As indicated from the foregoing figures, the outcomes of the viscoelastic model and the modified CHILE model conform to the outcomes presented by White and Kim [36]. Particularly, the excellent consistency between the viscoelastic model and the results presented by White and Kim was found. As illustrated in Figure 10, the final stress for the normal stress from the modified CHILE model is 24.5 MPa, which is 6.1% in excess of the viscoelastic solution, while the final stress for the transverse stress from the modified CHILE model in Figure 11 is 33 MPa, which is 9.1% in excess of the viscoelastic solution. The von Minses stress distribution of the composite laminate after cool-down, forecasted respectively by the modified and viscoelastic CHILE models, is illustrated in Figure 12.

### 5.3. Curing Deformation Analysis

**Figure 13:**
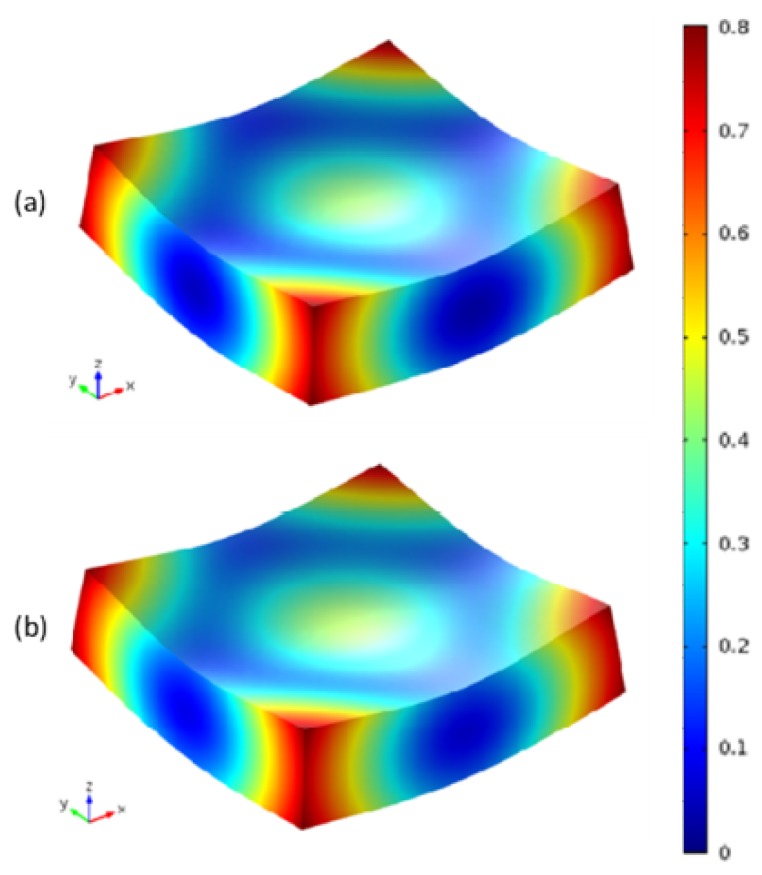
Contours of the deformation after cool-down forecasted by (**a**) Viscoelastic model, (**b**) Modified CHILE model.

This work introduces the Maximum deformation, defined as the maximum absolute displacement in the through-thickness direction, to present the gap of the deformation between the modified CHILE model and the viscoelastic CHILE model. The curing deformation contour forecasted by the two models after cool-down is indicated in Figure 13. Evidently, the laminate curves identically from four corners arising from the symmetrical and uniform laying of the fibers, as Figure 2 shows. The degree of deformation forecasted by the modified CHILE model is slightly higher than that of the viscoelastic model. The maximum deformation estimated by the modified CHILE model is 0.79 cm, which is 0.06 cm more than the viscoelastic model (0.73 cm).

### 5.4. Discussion

The previous sections discuss the differences between the viscoelastic model and the modified CHILE model. Besides forecast accuracy, a significant difference of computation time is also observed between the two models. In heat-up and hold periods, i.e., during curing, the modified CHILE model runs approximately 10 times faster than the viscoelastic model, while the multiple approximately reaches 20 times in the cool-down period. Clearly, the more complex the composite structure is, the more prominent the time benefit of the modified CHILE approach is.

## 6. Conclusions

In this paper, both the gold standard viscoelastic model and the modified CHILE model have been presented to forecast process-induced residual stress and deformation for the thermosetting resin composite laminates. The outcomes from these two kinds of fully 3D coupled simulation models are compared with each other to show their trade-offs. To improve the prediction accuracy, the evolution of the thermo-physical properties, with the temperature and DOC, is also considered.

This work establishes a four-layer composite laminate to validate two simulation models. A variable time parameter is introduced in the modified CHILE model to factor in relaxation features taken on by the materials. The outcomes show that the forecasted curing temperature, DOC, and remaining stress by the two proposed models are in good agreement with the results obtained by Kim and White. However, there is a significant difference in modulus related to elasticity between the modified and elastic CHILE models. Furthermore, the maximum deformation attained from the modified CHILE model is evidently in excess of that acquired by the viscoelastic model within acceptable limits.

Additionally, although the modified CHILE model has a lower accuracy than the viscoelastic model, the viscoelastic model, within the margin of error, can be replaced by the modified model in the numerical modeling of process-induced residual stress and deformation for a shorter computation time, and a far more efficient numerical implementation. As previously mentioned, in other generalized processing cases, the modified CHILE method was able to be adopted to delve into the impacts exerted by the thickness of the composite laminates, the stacking sequence, and the fiber volume fraction on curing-induced residual stress and deformation development.

## Figures and Tables

**Table 1:** Cure kinetic parameters of 3501-6 epoxy resin [1,2,33].

Parameter	Value	Parameter	Value
$A_{1}$ (min^−1^)	2.102 × 10^9^	$∆E_{1}$ (J/mol)	8.07 × 10^4^
$A_{2}$ (min^−1^)	−2.014 × 10^9^	$∆E_{2}$ (J/mol)	7.78 × 10^4^
$A_{3}$ (min^−1^)	1.96 × 10^5^	$∆E_{3}$ (J/mol)	5.66 × 10^4^
$H_{R}$ (J/Kg)	1.989 × 10^5^	R (J·mol^−1^·K^−1^)	8.3143

**Table 2:** Thermo-physical properties of AS4 carbon fiber and 3501-6 epoxy resin [1,2,33].

Property	Value
Resin density $\rho_{r}$ (mg·m^−3^)	$\begin{array}{c} 90\alpha +1232\left(\alpha \leq 0.45\right)\\ 1272\;\;\left(\alpha \geq 0.45\right) \end{array}$
Fiber density $\rho_{f}$ (mg·m^−3^)	1790
Resin specific heat capacity $C_{pr}$ (J·mol^−1^·K^−1^)	4184[0.468 + 5.975 × 10^−4^*T* − 0.141α]
Fiber specific heat capacity $C_{pf}$ (J·mol^−1^·K^−1^)	1390 + 4.50*T*
Thermal conductivity of resin $k_{r}$ (W·m^−1^·K^−1^)	0.04184[3.85 + (0.035*T* − 0.141)α]
Thermal conductivity of fiber $k_{f}$ (W·m^−1^·K^−1^)	0.742 + 9.02 × 10^−4^*T*

**Table 3:** Elastic mechanical properties of AS4/3501-6 prepreg constituents [1,2,26].

Property	AS4 Carbon Fiber	3501-6 Epoxy Resin
Longitudinal elastic modulus $E_{1}$ (Gpa)	206.8	3.2
Transverse elastic modulus $E_{2}=E_{3}$ (Gpa)	17.2	3.2
In-plane shear modulus $G_{12}=G_{13}$ (Gpa)	27.58	1.185
Transverse shear modulus $G_{23}$ (Gpa)	6.894	1.185
In-plane Poisson’s ratio $\upsilon_{12}=\upsilon_{13}$	0.2	0.35
Transverse Poisson’s ratio $\upsilon_{23}$	0.3	0.35
Longitudinal CTE $\phi_{1}$()	−9 × 10^−7^	5.76 × 10^−5^
Transverse CTE $\phi_{2}=\phi_{3}$()	7.2 × 10^−6^	5.76 × 10^−5^
Longitudinal CSE $\varphi_{1}$	0	−0.01695
Transverse CSE $\varphi_{2}=\varphi_{3}$	0	−0.01695

## Appendix A

This appendix presents micromechanical homogenization functions which are used to determine the overall composite properties of unidirectional lamina. Note that subscripts f and r represent fiber and resin, respectively. The thermo-physical properties taken on by composite materials, including the density, *ρ*, the specific heat capacity, *C_p_*, and the longitudinal thermal conductivity, *k_L_*, (*x*-direction in Equation (1)), can be respectively acquired by the following rule of mixtures [37]:

(A1) $$\rho =v_{f}\rho_{f}+\left(1-v_{f}\right)\rho_{r}$$

(A2) $$C_{p}=\frac{\rho_{f}v_{f}C_{pf}+\rho_{r}\left(1-v_{f}\right)C_{pr}}{\rho }$$

(A3) $$k_{L}=v_{f}k_{f}+\left(1-v_{f}\right)k_{r}$$

The transverse thermal conductivity taken on by composite materials, *k_T_*, (*y* and *z*-direction in Equation (1)) is able to be acquired in light of the E-S model [38]. It is established below:

(A4) $$\frac{k_{T}}{k_{r}}=\left(1-2\sqrt{v_{f}/\pi }\right)+\frac{1}{2B}\left[\pi -\left(4/\sqrt{1-\beta }\right)\tan^{-1}\left(\sqrt{1-\beta }/1+\beta \right)\right],$$

in which:

(A5) $$\beta =B^{2}v_{f}/\pi$$

(A6) $$B=\mu \left(k_{r}/k_{f}-1\right)$$

(A7) μ=a/b,

where a and b respectively denote the axial lengths of the elliptic section of the fiber along the y-axis and z-axis. The cross section of the fiber is round in this paper. Accordingly a equals to b, i.e., u=1.

This appendix lists the self-consistent micromechanics homogenization functions [36] adopted to ascertain the properties taken on by composite. The principal directions of unidirectional lamina are represented by subscripts 1, 2, and 3. The Young’s modulus taken on by composite materials in the longitudinal direction of the fiber is denoted below:

(A8) $$E_{1}=E_{1f}v_{f}+E_{r}\left(1-v_{f}\right)+\frac{4{\left(\upsilon_{r}-\upsilon_{12f}\right)}^{2}K_{r}K_{f}G_{r}v_{f}\left(1-v_{f}\right)}{\left(K_{f}+G_{r}\right)K_{r}+\left(K_{f}-K_{r}\right)G_{r}v_{f}},$$

in which:

(A9) $$K_{f}=\frac{E_{1f}E_{2f}}{2\left(1-\upsilon_{23f}\right)E_{1f}-4\upsilon_{12f}^{2}E_{2f}},$$

(A10) $$K_{r}=\frac{E_{r}}{2\left(1-\upsilon_{r}\right)-4\upsilon_{r}^{2}},$$

where $K_{r}$ and $K_{f}$ denote the bulk modulus of the resin and fiber, respectively; $\upsilon_{r}$ denotes the Poisson’s ratio of the resin; $\upsilon_{12f}$ and $\upsilon_{23f}$ denote the in-plane Poisson’s ratio and transverse Poisson’s ratio of the fiber; $G_{r}$ denotes the shear modulus of the resin; $E_{r}$ denotes the Young’s modulus of the resin; $E_{1f}$ and $E_{2f}$ denote the Young’s modulus of the fiber transverse direction and in the longitudinal direction.

The in-plane shear modulus taken on by composite materials is denoted below:

(A11) $$G_{12}=G_{13}=G_{r}\frac{\left(G_{12f}+G_{r}\right)+\left(G_{12f}-G_{r}\right)v_{f}}{\left(G_{12f}+G_{r}\right)-\left(G_{12f}-G_{r}\right)v_{f}}$$

The out-of-plane shear modulus taken on by composite materials is denoted below:

(A12) $$G_{23}=G_{r}\frac{\left(G_{23f}+G_{r}\right)K_{r}+2G_{23f}G_{r}+\left(G_{23f}-G_{r}\right)K_{r}v_{f}}{\left(G_{23f}+G_{r}\right)K_{r}+2G_{23f}G_{r}-\left(G_{23f}-G_{r}\right)\left(K_{r}+2G_{r}\right)v_{f}}$$

where $G_{23f}$ and $G_{12f}$ are the out-of-plane shear modulus and in-plane shear modulus of the fiber.

The plane strain bulk modulus taken on by composite materials is denoted below:

(A13) $$K_{2}=\frac{\left(K_{f}+G_{r}\right)K_{r}-\left(K_{f}-K_{r}\right)G_{r}v_{f}}{\left(K_{f}+G_{r}\right)-\left(K_{f}-K_{r}\right)v_{f}}$$

The Young’s modulus taken on by composite materials in the transverse direction of the fiber is denoted below:

(A14) $$E_{2}=E_{3}=\frac{1}{1/4K_{2}+1/4G_{23}+\upsilon_{12}^{2}/E_{1}}$$

The in-plane Poisson’s ratio taken on by composite materials is denoted below:

(A15) $$\upsilon_{12}=\upsilon_{13}=\upsilon_{12f}v_{f}+\upsilon_{r}\left(1-v_{f}\right)+\frac{\left(\upsilon_{r}-\upsilon_{12f}\right)\left(K_{r}-K_{f}\right)G_{r}v_{f}v_{r}}{\left(K_{f}+G_{r}\right)K_{r}+\left(K_{f}-K_{r}\right)G_{r}v_{f}}$$

The out-of-plane Poisson’s ratio taken on by composite materials is denoted below:

(A16) $$\upsilon_{23}=1-\frac{E_{1}E_{2}+4\upsilon_{12}^{2}E_{2}K_{2}}{2E_{1}K_{2}}$$

The longitudinal CTE taken on by composite materials is denoted below:

(A17) $$\phi_{1}=\frac{v_{f}\phi_{1f}E_{1f}+\left(1-v_{f}\right)\phi_{m}E_{1m}}{v_{f}E_{1f}+\left(1-v_{f}\right)E_{1m}}$$

The transverse CTE taken on by composite materials is denoted below:

(A18) $$\phi_{2}=\phi_{3}=v_{f}\left(\phi_{2f}+\upsilon_{12f}\phi_{1f}\right)+v_{m}\left(1+\upsilon_{m}\right)\phi_{m}$$

(A19) $$-\left(\upsilon_{12f}v_{f}+\upsilon_{m}v_{m}\right)\frac{\phi_{1f}E_{1f}v_{f}+\phi_{1m}E_{1m}v_{m}}{E_{1f}v_{f}+E_{1m}v_{m}}$$
